# Cannabis use and cognitive impairment in schizophrenia

**DOI:** 10.1192/j.eurpsy.2022.1158

**Published:** 2022-09-01

**Authors:** M. Gonçalves, J. Romão, R. André, F. Félix, G. Andrade, R. Saraiva, E. Dornelles, E. Fernandes, M. Abreu, I. Chendo, F. Ismail

**Affiliations:** Centro Hospitalar Universitário Lisboa Norte, Psychiatry, Lisboa, Portugal

**Keywords:** cognitive impairment, schizophrénia, Cannabis use

## Abstract

**Introduction:**

Neurocognitive deficits amongst patients with schizophrenia are considered one of schizophrenia’s central features. These deficits appear to be present from the first episode of psychosis (FEP) and certain cognitive impairments could be components of a genetic vulnerability to schizophrenia. Regarding research on cannabis and cognition in schizophrenia, different studies have assessed neurocognitive functions: memory, attention/vigilance, processing speed, verbal learning, executive functions, and verbal fluency.

**Objectives:**

The aim is to do a review of recent findings concerning the association of cannabis use with cognition in schizophrenia.

**Methods:**

A literature review was conducted using the PubMed search database.

**Results:**

Patients with schizophrenia and concomitant cannabis use are associated with worse performance in immediate verbal learning, and in some studies with worse working memory performance. There is an improvement of verbal memory when they cease the cannabis’ consumption. Regarding attention capacity and memory types assessed, the results are controversial. In FEP, heavy cannabis use during the previous year correlates with slower processing speed. Also, FEP-patients with cannabis use but no family history of psychosis perform worse in executive functions, while those with a family history of psychosis perform better.

**Conclusions:**

The studies of psychosis, cannabis and cognition differ in relevant aspects, which might be connected to the result variability. Therefore, before solid conclusions can be reached, it is important to carry out longitudinal studies to understand the changes in the cognitive variables, which can depend on the pattern of cannabis’ use (concurrent or prior to the FEP). Possible confounding variables that might be present should be acknowledged.

**Disclosure:**

No significant relationships.

